# Ultra-Fine Bainite in Medium-Carbon High-Silicon Bainitic Steel

**DOI:** 10.3390/ma17102225

**Published:** 2024-05-09

**Authors:** Xinpan Yu, Yong Wang, Huibin Wu, Na Gong

**Affiliations:** 1 Collaborative Innovation Center of Steel Technology, University of Science and Technology Beijing, Beijing 100083, China; xinpan_yu@163.com; 2 School of Materials Science and Engineering, Nanyang Technological University, Singapore 639798, Singapore; 3 Institute of Materials Research and Engineering (IMRE), A*STAR (Agency for Science, Technology, and Research), 2 Fusionopolis Way, Singapore 138634, Singapore

**Keywords:** prior martensite, transformation behavior, retained austenite, mechanical property

## Abstract

The effects of austenitizing and austempering temperatures on the bainite transformation kinetics and the microstructural and mechanical properties of a medium-carbon high-silicon ultra-fine bainitic steel were investigated via dilatometric measurements, microstructural characterization and mechanical tests. It is demonstrated that the optimum austenitizing temperature exists for 0.3 wt.%C ultra-fine bainitic steel. Although the finer austenite grain at 950 °C provides more bainite nuclei site and form finer bainitic ferrite plates, the lower dislocation density in plates and the higher volume fraction of the retained austenite reduces the strength and impact toughness of ultra-fine steel. When the austenitizing temperature exceeds 1000 °C, the true thickness of bainitic ferrite plates and the volume fraction of blocky retained austenite in the bainite microstructure increase significantly with the increases in austenitizing temperature, which do harm to the plasticity and impact toughness. The effect of austempering temperature on the transformation behavior and microstructural morphology of ultra-fine bainite is greater than that of austenitizing temperature. The prior martensite, formed when the austempering temperature below *M*s, can refine the bainitic ferrite plates and improve the strength and impact toughness. However, the presence of prior martensite divides the untransformed austenite and inhibits the growth of bainite sheaves, thus prolonging the finishing time of bainite transformation. In addition, prior martensite also strengthens the stability of untransformed austenite though carbon partition and enhances the volume fraction of blocky retained austenite, which reduces the plasticity of ultra-fine bainitic steel. According to the experimental results, the optimum austempering process for 0.3 wt. %C ultra-fine bainitic steel is through austenitization at 1000 °C and austempering at 340 °C.

## 1. Introduction

Ultra-fine bainite steel, also known as super bainite steel, nano-bainite steel and low-temperature bainite steel, has become a research hotspot due to its excellent combination of strength and ductility [1,2,3]. Ultra-fine bainitic ferrite plates, 20 nm~1 μm in thickness, within high dislocation densities provide the steel with high strength, and the film-like retained austenite embedded into the plates can absorb microcrack propagation, thereby ensuring its high toughness property [4,5,6]. The impressive combination of ultimate tensile strength (>2 GPa) and ductility (>15%) has been achieved latterly in medium-carbon bainitic steel, indicating that the ultra-fine bainitic steel has huge potential in the field of wear-resistant components such as gears, bearing, and so on [6,7,8,9].

The first generation of ultra-fine bainitic steel with high carbon content was obtained though austempering at low temperature (125–325 °C) from 2 to 90 days [10]. With further research about the bainite transformation mechanism and the relationship between microstructure and mechanical properties, the bainite transformation process is clearly accelerated by the following methods: (i) Through adjusting the chemical component design [11,12,13], the segregation of boron at prior austenite grain boundaries can retard the bainite transformation at low temperature [13]. (ii) By increasing the nucleation sites though lowering the direct austempering temperature to below *M*s [14,15,16,17], the supercooled austenite is deformed [18,19,20,21] and partial bainite/athermal martensite is obtained before bainite transformation [22,23,24,25,26]. However, there are quite a few research studies about the effect of prior austenite on bainite transformation, and the results are controversial. On the one hand, Seok-Jae Lee [27] and Jing Zhao et al. [28] thought that the bainite transformation kinetics of medium-carbon steel increased as the austenite grain size decreased. On the other hand, F Hu et al. [29] noticed that the coarser austenite grains provide less nucleation sites, which are beneficial to bainite growth. That is to say, there is an optimum austenitizing temperature for ultra-fine bainite steels with different carbon contents.

The austempering temperature below *M*s was selected to accelerate the transformation kinetics and enhance the mechanical properties of ultra-fine bainite steel though forming partial martensite in recent research [30,31,32]. Although the presence of prior martensite can increase the nucleation sites of bainite in the following isothermal process, the formation of the former phase also refines the untransformed austenite, and then inhibits the growth of bainitic ferrite plates [33,34]. In addition, the diffusion of carbon ejected from super-saturated martensite to austenite increases the stability of the latter phase, thereby reducing the degree of bainite transformation [35,36]. Therefore, it is necessary to contrastively analyze the effects of austempering temperatures above and below *M*s on transformation kinetics and microstructural morphology to obtain the ultra-fine bainite steel with optimal mechanical properties.

In order to determine the effect of austenitizing temperature and austempering temperature on ultra-fine bainitic steel, a medium-carbon steel with 0.30 wt.%C was selected for research in the paper. A series of heat treatment processes with different austenitizing temperatures and austempering temperatures were carried out to explore the relationship between the heat treatment processes and bainite transformation kinetics, microstructural morphology, and mechanical properties of medium-carbon steel. The multiphases of martensite, bainitic ferrite with different volume fractions and dislocation densities, and the retained austenite with different morphologies and carbon concentrations along with their mechanical properties were observed and measured though scanning electronic microscopy (SEM), transmission electron microscope (TEM), X-ray diffractometer (XRD) and tensile testing.

## 2. Experimental Procedures

### 2.1. Steel Composition and Thermomechanical Treatments

The chemical composition of the tested steel used in this paper was 0.30C-1.40Si-1.50Mn-1.18Cr-1.15Al-0.49Mo-0.61Ni-0.019Nb-0.0026B (wt.%). Si can inhibit the precipitation of cementite during isothermal transformation, enhance the thermal stability of retained austenite and accelerate bainite transformation at low temperature [37,38]. The addition of Mn and Cr can increase the hardenability of medium-carbon steel [39,40]. Al can increase the driving force of austenite to ferrite (Δ*G* ^γ→α^) [41,42]. The precent of Nb and Mo can refine the prior austenite grains and accelerate the following bainite transformation [11]. The boron segregation at prior austenite can affect grain size and determine the nucleation site and transformation kinetics of bainite [12]. The tested steel was smelted in a vacuum furnace and forged into a block measuring 60 × 80 × 100^3^ mm. Samples with a size of φ4 × 10 mm^3^ was cut and used for measuring the transformation temperature and kinetics of the tested steel with different heat treatment processes. Samples with a size of 12 × 60 × 60 mm^3^ composed of sodium nitrate and potassium nitrite (1:1 in weight) were cut and used for the subsequent heat treatments in a muffle furnace and a salt bath furnace.

**Figure 1:**
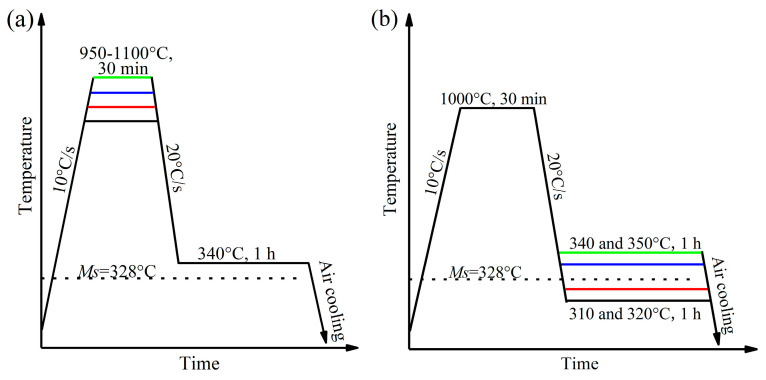
Heat treatment program to study the effect of austenitizing temperature (**a**) and austempering temperature (**b**) on bainite transformation.

The *M*s temperature was measured to be 328 °C by the DIL 805A thermal dilatometer (Waters, Milford, MA, USA). The heat treatment processes are given in Figure 1. As shown in Figure 1a, the samples were fully austenitized at 950–1100 °C about 30 min with a heating rate of 10 °C/s, then cooled to 340 °C for 1 h and finally quenched into room temperature to research the effect of austenitizing temperature on bainite transformation, the microstructure, and mechanical properties of the tested steel. Similarly, after austenitizing at 1000 °C for 30 min, the samples were quenched to below *M*s (310 and 320 °C) and above *M*s (340 and 350 °C) about 1 h to research the effect of austempering temperatures, as given in Figure 1b. The dilatation of the samples along the radial direction was recorded and used to characterize the bainite transformation kinetics.

### 2.2. Microstructural Characterization

SEM samples were mechanically ground, polished and etched with a 4% nital solution. The morphological characters of prior austenite grain, bainite and retained austenite were observed at low magnification though SEM (ZEISS EVO 18) (Carl Zeiss, Oberkochen, Germany). TEM samples were thinned to ~50 μm by a precision ion polishing system (Gatan 691) (Gatan, Pleasanton, CA, USA). The sizes of the bainitic ferrite were measured and counted using Tecnai F20 (FEI, Hillsboro, OR, USA), operating at 200 kV. The dislocation density within bainitic ferrite and the volume fraction and average carbon content of retained austenite were analyzed by XRD with Cu-*Ka* radiation operating at 40 kV and 40 mA. The XRD pattern was drawn based on the data obtained at a scanning rate of 1°/min and 2θ range of 30–100°. The dislocation density of bainitic ferrite (*ρ*) was counted according to the Williamson–Hall formula, as presented in Equation (1) [43]. The volume fraction of retained austenite (*V*_γ_) was calculated based on the integrated intensities of (200), (220) and (311) austenite peaks and (200), (211) ferrite peaks [44]. The volume fractions of prior martensite (*V*_M_) within the samples austempered below *M*s are calculated according to Equation (2). Finaly, the volume fraction of bainitic ferrite, *V*_B_, is calculated by Equation (3). The average carbon content of retained austenite (*C*_γ_) was determined though Equation (4) [45]. Carbon contents of retained austenite with film-like and blocky morphology were calculated by the Gaussian multi-pears fitting method, as reported in the literature [46,47].

*ρ* = 14.4 *e*^2^/b^2^ (1)

*V*_M_ *=* 1 − *e*^−0.011(*M*s-QT)^ (2)

*V*_B_ = 1 − *V*_γ_ − *V*_M_ (3)

*C*_γ_ = (*a* − 3.578)/0.033 (4)

where *M*s and QT are the martensite start temperature and the directly austempering temperature, respectively, of the tested sample; *e* is the micro-strain measured by MDI Jade 6.0 software (6.0, Materials Data, Livermore, CA, USA); b is the Burgers vector of ferrite (0.248 nm); and *a* is the austenite lattice parameter in Å.

### 2.3. Mechanical Testing

In accordance with GB/T228.1-2021, plate samples with a parallel length of 52 mm and a cross-section of 15 mm × 4 mm were used for tensile tests, which were conducted using the MTS-CMT5105 universal testing machine (Millennium Technology Services, San Francisco, CA, USA), with a loading rate of 3 mm/min. Standard Charpy V-notch samples, with dimensions of 10 mm × 10 mm × 50 mm, were used to measure the impact toughness at room temperature using the JBDW-300D impact tester (Jinan Kehui test equipment Co., LTD, Jinan, China). For each heat treatment process, three samples were tested. 

## 3. Results

### 3.1. Transformation Kinetics

**Figure 2:**
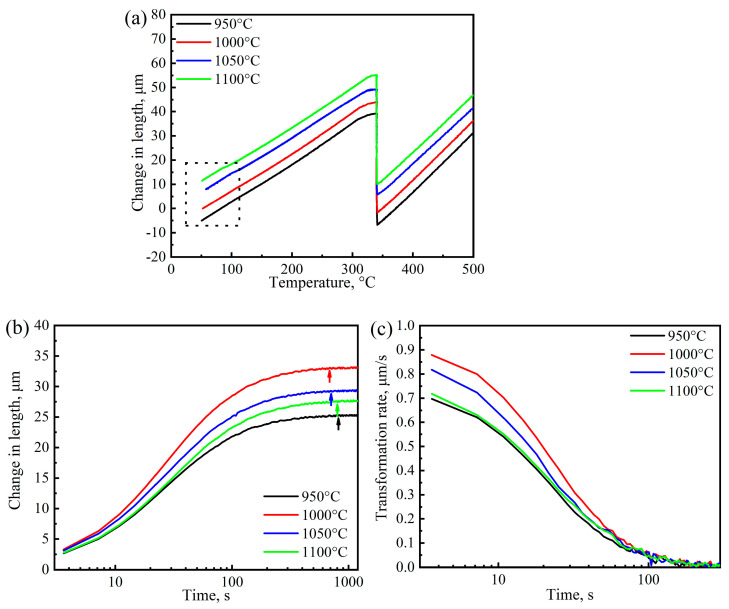
Dilatation–temperature (**a**), dilatation–time (**b**) and transformation rate–time (**c**) curves of the samples during holding at 340 °C directly from different austenitizing temperatures.

Figure 2 shows the bainite transformation kinetics curves, including dilatation–temperature curves, dilatation–time curves, and transformation rate–time curves of the samples at different austenitizing temperatures. The cooling rate from austenitizing stage down to isothermal stage is large enough to avoid the formation of ferrite and pearlite, so the starting temperature of the dilatation–temperature curve of the samples was chosen to be 500 °C, as shown in Figure 2a. Temperature-independent bainite formation occurs immediately when the austempering temperature reaches 340 °C. During further cooling to room temperature, there is no obvious deviation of the dilatation–temperature curves, indicating that the blocky retained austenite does not decompose into martensite, as shown by the dashed box in Figure 2a. The dilatation–temperature curves at the austempering stage were normalized to obtain the dilatation–time curves, as shown in Figure 2b. There is no obvious transformation incubation time for all the samples at different austenitizing temperatures. The completion time of isothermal bainite transformation was determined though the tangent method. The isothermal bainite completion times of the samples at 950–1100 °C were 820.2 s, 687.0 s, 708.6 s and 802.2 s. The transformation rate–time curves were obtained by derivation of the dilatation–time curves, as shown in Figure 2c. At the beginning of the isothermal stage, the bainite transformation rate reaches the maximum value, and then decreases gradually as the isothermal time increases. It is worth mentioning that the transformation rate of the sample austenitized at 1000 °C is higher than those of the samples which were austenitized at 950 °C, 1050 °C and 1100 °C.

**Figure 3:**
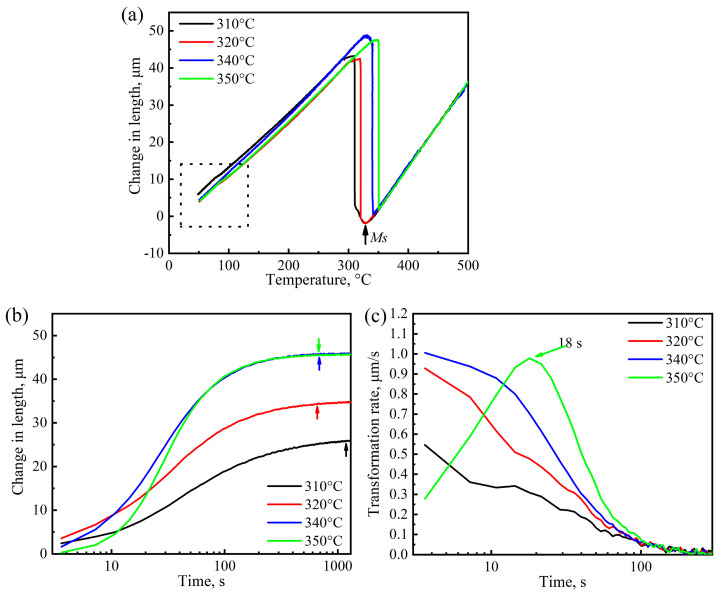
Dilatation–temperature (**a**), dilatation–time (**b**) and transformation rate–time (**c**) of the samples during holding at different austempering temperatures directly from 1000 °C.

The bainite transformation kinetic curves of the samples austempering at different temperatures are shown in Figure 3. When the isothermal bainite transformation temperatures were 310 and 320 °C, as shown in Figure 3a, the dilatation–temperature curves of the samples deviated slightly after the temperature drops below *M*s, indicating that the prior martensite formed before isothermal bainite transformation. The lower the isothermal bainite transformation temperature, the higher the volume fraction of prior martensite formation [16,17,18,19]. The dilatation–time curves and bainite transformation rate–time curves of the sample austempered at different temperatures were obtained similarly with those in the case of samples austenitized at different temperatures. The results are shown in Figure 3b,c. The transformation incubation times of the samples austempered at 310–350 °C are also not obvious like those of the samples austenitized at 950–1100 °C. Except for the samples austempered at 310 °C, the bainite transformation completion times of the samples austempered at 320 °C, 340 °C and 350 °C are not different, which are 658.8 s, 687.0 s and 678.8 s, respectively. Although a larger degree of supercooling provides higher transformation dynamic, and more α/γ interfaces provide nucleation sites for bainite transformation, the transformation completion time of the sample austempered at 310 °C is 1184.4 s, which is significantly longer than in the case of the other austempered samples. The maximum transformation rate increases with the increase in austempering temperature, and decreases after reaching the peak value at 340 °C. It is worth noting that the maximum transformation rate of the samples austempered at 350 °C appears 18 s after the beginning of the bainite transformation, while the other austempered samples have no obvious transformation incubation period, and the maximum transformation rate occurs at the beginning of bainite transformation.

### 3.2. Microstructure

**Figure 4:**
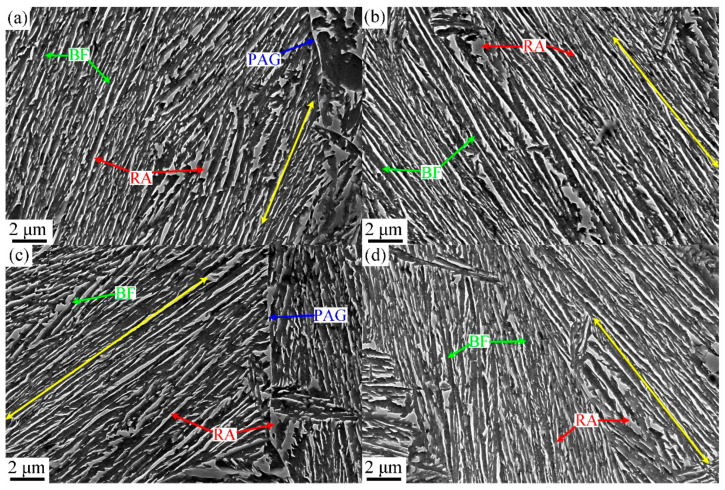
SEM micrographs of the samples austenitized at different temperatures: (**a**) 950 °C, (**b**) 1000 °C, (**c**) 1050 °C, (**d**) 1100 °C.

**Figure 5:**
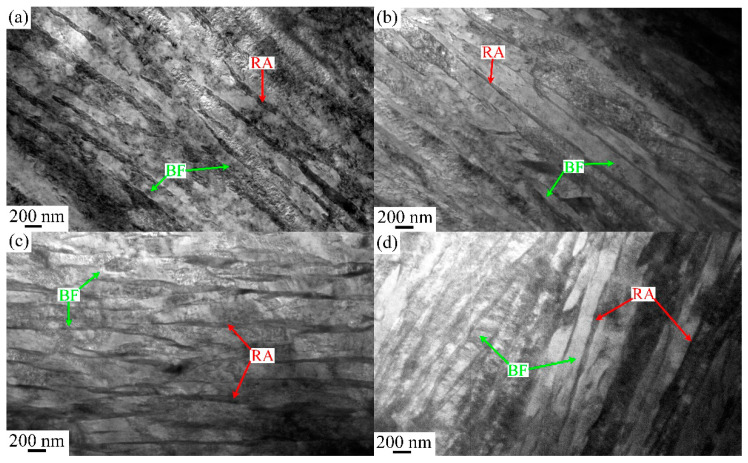
TEM micrographs of the samples austenitized at different temperatures: (**a**) 950 °C, (**b**) 1000 °C, (**c**) 1050 °C, (**d**) 1100 °C.

**Figure 6:**
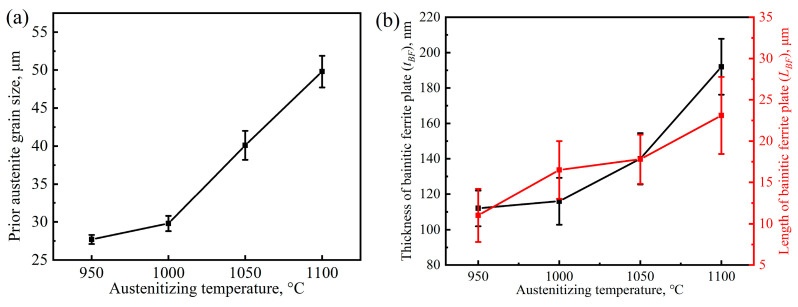
Grain size of prior austenite (**a**) and thickness and length of bainitic ferrite plate (**b**) changing with the austenitizing temperature.

**Figure 7:**
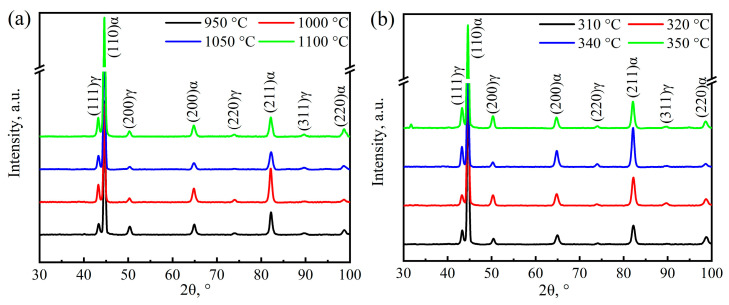
XRD patterns of the samples after different heat treatments: (**a**) austenitizing temperature; (**b**) austempering temperature.

The microstructural macroscopic morphology of ultra-fine bainite within the sample under different isothermal transformation process parameters are shown in Figure 4, Figure 5, Figure 6 and Figure 7. It can be observed that the microstructure of the samples after different austempering processes is the same, which is composed of bainitic ferrite plates and retained austenite, in which the retained austenite shows film-like and blocky morphology. The microstructural composition and the morphology of ultra-fine bainite from the samples austenitized at different temperatures are shown in Figure 4 and Figure 5, respectively. The ultra-fine bainitic ferrite plates nucleate at the prior austenite grain boundary (PAG) or α/γ interface, and grow into the grain interior. In order to measure the size of prior austenite grain size, samples were directly quenched into water from different austenitizing temperatures, and the PAG was etched using an electrochemical etching method [27]. The equivalent grain size of prior austenite in the tested sample is smaller than 30 μm after austenitizing at 950 and 1000 °C, and increases significantly when the austenitizing temperature exceeds 1000 °C (Figure 6a). It can be seen from the SEM micrographs that the size of blocky retained austenite and the length of bainitic ferrite plate (*L_BF_*) increase with an increment in austenitizing temperate. To analyze quantitatively the effect of austenitizing temperature on the microstructure of ultra-fine bainite, the average length and true thickness of bainitic ferrite were measured 100 times based on SEM and TEM micrographs. The true thickness of bainitic ferrite (*t_BF_*) can be obtained according the following equation:

*t_BF_* = 2*L_T_*/π (5)

where *L_T_* presents the mean linear intercept, which is determined along the normal direction of the bainitic ferrite plates. The statistical results are compared and listed in Figure 6b. The value of *L_BF_* increases linearly with the austenitizing temperature from 950 °C to 1100 °C, but the rule does not apply to the case of *t_BF_*. The *t_BF_* increases slowly when the austenitizing temperature increases from 950 °C to 1000 °C. However, the *t_BF_* increases significantly when the austenitizing temperature exceeds 1000 °C. The strength of ultra-fine bainite steel depends on the true thickness of bainitic ferrite plates, and the finer plates, the higher strength of the tested steel.

**Figure 8:**
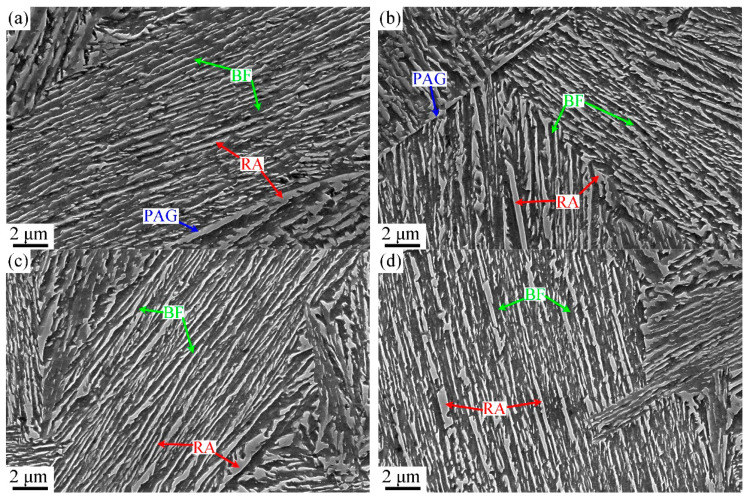
SEM micrographs of the samples austempered at different temperatures: (**a**) 310 °C, (**b**) 320 °C, (**c**) 340 °C, (**d**) 350 °C.

The XRD patterns of the samples austenitized at different temperatures are shown in Figure 8a. the microstructure of the samples is composed of ferrite phase (α) and austenite phase (γ), and no diffraction peak of carbide. The volume fractions and average carbon contents of retained austenite were counted and are listed in Table 1. With the increase in austenitizing temperature, the volume fraction of retained austenite within the samples first decreases and then increases. The volume fraction of retained austenite reaches the lowest value of 8.8 vol.% from the sample austenitized at 1000 °C. However, the average carbon content of the retained austenite shows an opposite change, and the average carbon content of the retained austenite reaches the peak value of 1.9% when the austenitizing temperature is selected as 1000 °C. The carbon content of retained austenite is closely related to the plasticity and toughness of ultra-fine bainitic steel [48]. Based on the Gaussian multi-peak fitting method, (200) austenite peak was selected to analyze the volume fraction and carbon content of film-like and blocky retained austenite within the tested steel [6]. The corresponding calculation results are also shown in Table 1. The carbon contents of film-like retained austenite in the samples after austenitizing at 1000 and 1050 °C are significantly higher than those of other samples. However, the carbon content of blocky retained austenite has little correlation with the austenitizing temperature, which may be related to the isothermal bainite transformation process of supercooled austenite.

**Figure 9:**
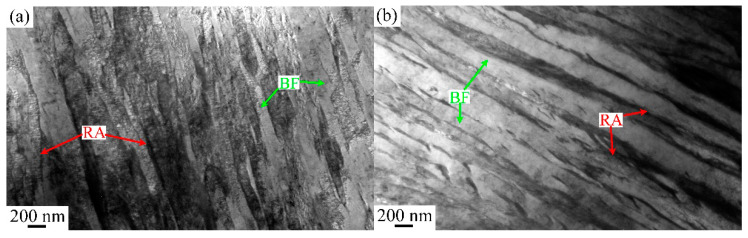

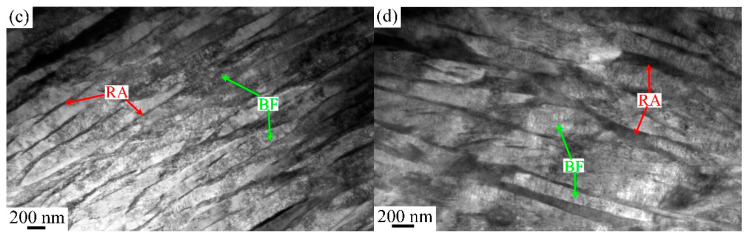
TEM micrographs of the samples austempered at different temperatures: (**a**) 310 °C, (**b**) 320 °C, (**c**) 340 °C, (**d**) 350 °C.

**Figure 10:**
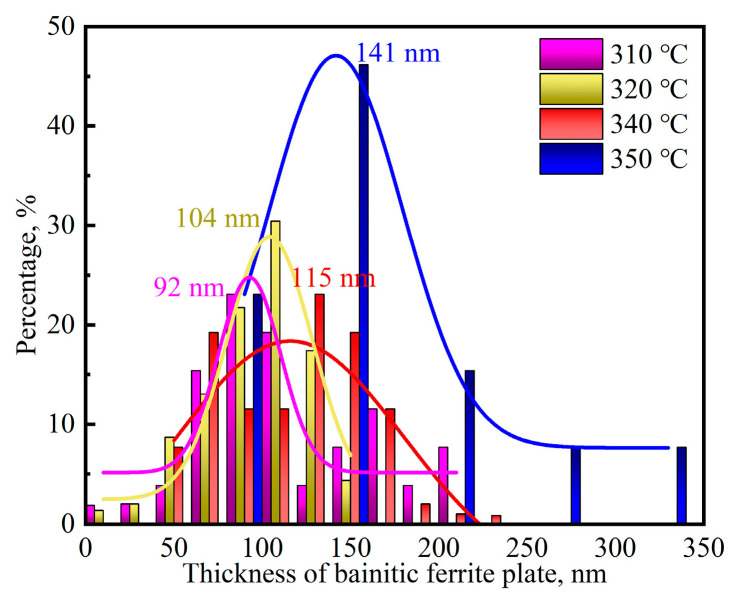
True thickness of bainitic ferrite plate from the samples austempered at different temperatures.

The ultra-fine bainite microstructural morphology from the samples austempered at different temperatures is shown in Figure 8 and Figure 9. Like the case of that austenitized at different temperatures, the microstructural parameters of the tested steel were also counted and are shown in Figure 10. When the austempered temperature is below *M*s, specifically 310 °C, the true thickness of bainitic ferrite plates within the samples is significantly finer than those of the samples whose austempered temperatures are above *M*s. The lower the austempered temperature, the finer the bainitic ferrite plate, which can be explained by the fact that prior martensite in supercooled austenite provides more nucleation sites for the following bainite transformation.

**Figure 11:**
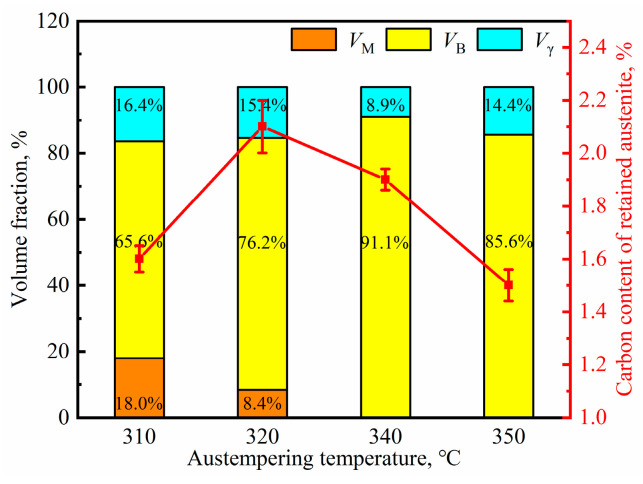
Volume fraction of constituting phases and carbon content of retained austenite from the samples austempered at different temperatures. VM: prior martensite, VB: bainitic ferrite, Vγ: retained austenite.

The XRD pattern of the samples austempered at different temperatures is shown in Figure 8b, and the volume fraction of prior martensite, bainitic ferrite, retained austenite and the average carbon content within the latter phase of the samples is calculated and shown in Figure 11. The constitution of the microstructures of the samples are different when the austempering temperature is below or above *M*s. For the samples austempered below *M*s, although the volume fraction of retained austenite changes little, the average carbon content of retained austenite decreases significantly. However, the volume fraction of retained austenite increases with an increment in temperature, and the average carbon content within it changes in the opposite way when the austempering temperature is above *M*s. The calculated results of retained austenite with film-like and blocky morphology and their carbon content based on the Gaussian multi-pears fitting method are listed and compared in Table 2. On the whole, like the case of the effect of austenitizing temperature, the austempering temperature shows little effect on the carbon content of blocky retained austenite. Although the volume fraction of blocky retained austenite (*V*_B-RA_) increases significantly for the sample austempered below *M*s, more carbon atoms are injected into film-like retained austenite. When austempering temperature exceeds *M*s, both the volume fractions of the two morphologies of the retained austenite increase with an increment in austempering temperature, but the carbon content of the film-like retained austenite decreases, as shown in Table 2.

### 3.3. Mechanical Properties

**Figure 12:**
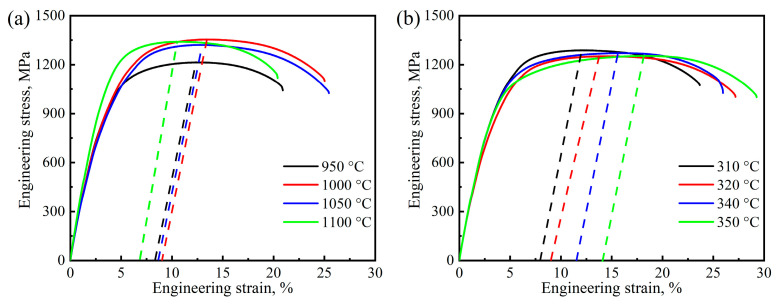
Engineering stress–strain curves of the tested steel after different heat treatments: (**a**) austenitizing temperature; (**b**) austempering temperature.

**Figure 13:**
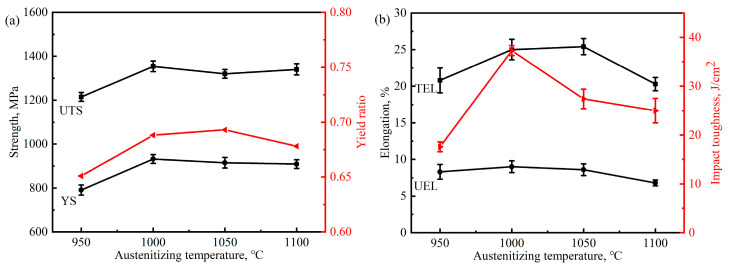
Mechanical property parameters of the samples after austenitizing at different temperatures: (**a**) strength and yield ratio, (**b**) elongation and impact toughness.

The engineering stress–strain curves of the samples austenitized at various temperatures are shown in Figure 12a. As observed, the engineering stress–strain curves of the samples austenitized at different temperatures do not have much difference except when austenitizing at 950 °C. The value of the yield strength (YS), ultimate tensile strength (UTS), yield ratio, uniform elongation (UEL), total elongation (TEL) and impact toughness at room temperature of different austenitized samples are summarized and shown in Figure 13. The UTS and TS are higher in the samples austenitized at 1000–1100 °C (~1300 MPa and 900 MPa, respectively) compared to the sample austenitized at 950 °C (1200 MPa and 800 MPa). Although the value of *t*_BF_ of the 950 °C sample is close to that of the 1000 °C sample, the volume fraction of bainitic ferrite and the dislocation density within are probably due to the increase in strength. The UEL of different austenitized samples exhibit a weaker correlation with austenitizing temperature, while the TEL and impact toughness of austenitized samples increase firstly and then decrease with the increase in austenitizing temperature. The austenitized samples have lower impact toughness and show brittle behavior under room temperature; however, the 1000 °C austenitized sample exhibits an extraordinary impact toughness of approximately 25 J. By contrast, the 950 °C austenitized sample only absorbs half of this value at the same impact testing.

**Figure 14:**
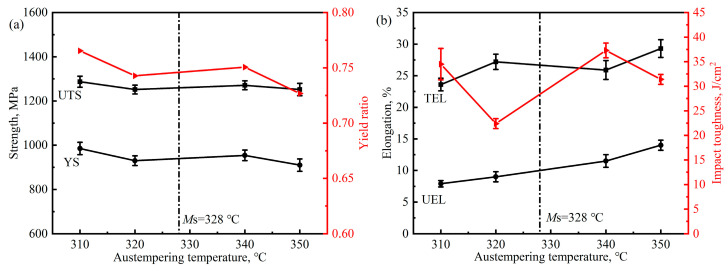
Mechanical property parameters of the samples after austempering at different temperatures: (**a**) strength and yield ratio, (**b**) elongation and impact toughness.

Figure 12b shows the engineering stress–strain curves of the samples austempered at above and below *M*s. Unlike the case of austenitized samples, the tensile curves of the samples, austempered at 310 °C, 320 °C (below *M*s) and 340 °C, 350 °C (above *M*s), are not obviously different, but their strengths and elongation are varied. The parameters of tensile property and impact toughness of the austempered samples are summarized and compared in Figure 14. It can be seen that the austempering temperature shows little effect on the UTS of the samples. When the austempering temperature is above *M*s, the value of YS and impact toughness of the samples decrease, while the elongation increase with the increase in austempering temperature. The same phenomenon also occurs when the austempering temperature is below *M*s. This may be related to the volume fraction of bainite and prior martensite as well as the mechanical stability of retained austenite. The microstructure of the samples is composed of bainitic ferrite and retained austenite when austempered at 340 and 350 °C. Their mechanical properties depend on the volume fraction of bainite ferrite and the thickness of the plates as well as the mechanical stability of the residual austenite. After austempering at 310 and 320 °C, the prior martensite formed in the microstructure of the samples, and the lower the austempering temperature, the higher the volume fraction of prior martensite. The presence of prior martensite not only change the composition of the samples, but also refine the thickness of bainitic ferrite plate and decrease the average carbon content of retained austenite. The phases and corresponding microstructure parameter on the mechanical properties of the austempered samples will be discussed in Section 4.

## 4. Discussion

In the process of manufacturing ultra-fine bainitic steel by conventional one-step austempering, the transformation kinetics, microstructural morphology and mechanical properties of bainite mainly depend on the driving force of bainite transformation and the nuclei site for bainitic ferrite plates [49]. In the present paper, the effects of transformation temperature on bainite finishing time, the thickness of bainitic ferrite plates and the density dislocation within it, the volume fraction of retained austenite and carbon content within it, and the tensile and impact properties of the tested steel at different austenitizing and austempering temperatures were all analyzed to explore the optimal one-step isothermal quenching process.

At the same austempering temperature, which is above *M*s, bainitic ferrite mainly nucleates at the grain boundaries of prior austenite (PAG) or the bainite sheaves formed in the early transformation, and grows into untransformed austenite grain (Figure 1). After austenitizing at 950 °C, finer austenite grains are formed in the tested steel. Compared to that in the samples austenitized at higher temperature (e.g., 1000 °C), the proportion of PAG in the sample is higher, which means that more bainite sheaves can be formed within a single austenite grain when austempering at the same temperature. In addition, more nucleation sites also mean that the incubation period of bainite transformation is very short, which can be reflected in the transformation rate curves that the perk value of transformation rate reached in the beginning of bainite transformation (Figure 2c). Due to the formation of the prior bainite sheaves in austenite grain, the growth of the newly formed bainite lath in the untransformed austenite region is inhibited, which results in a low transformation rate and a longer transformation finish time.

Another result of the more bainite nuclei sites is that the bainitic ferrite plates is refined after the transformation is completed, which improves the strength of the tested steel. The thickness of the plates at 950 and 1000 °C is comparable, but the difference in yield strength between them is 141 MPa, which can be attributed to the dislocation density (3.3 × 10 ^15^ m^−2^ to 5.4 × 10 ^15^ m^−2^) within the bainitic ferrite. The growth of bainite in the 950 °C austenitized sample is inhibited, resulting in retained austenite with high volume fraction in the final microstructure, especially the blocky retained austenite (8.6 vol.%). The blocky retained austenite with low carbon content tends to transform to martensite during the tensile and impact process, which is harmful to the plasticity and toughness of the tested steel. As a result, the impact toughness of the 950 °C sample is only 17.6 J, which is consistent with the results for the previous study [50].

After the austenitizing temperature exceeds 1000 °C, with the increase in austenitizing temperature, the prior austenite grain size increases obviously, but the ratio of grain boundary in tested steel decreases and then reduces the bainite nuclei sites. Nonetheless, the growth inhibition of bainitic ferrite plates is weaker, and the length of the plates increases significantly (Figure 6). After the austenitizing temperature is further increased to 1100 °C, the prior austenite grain size increases significantly, which can be reflected by the length of bainite ferrite plates after the transformation is completed. Although the highest degree of supercooling can provide the highest driving force for following bainite transformation, the proportion of PAG acted as the bainite nuclei site is lower compared with that in the 1000 °C austenitized sample, leading to the thicker bainite ferrite plates formed in the final microstructure (Figure 5d). In this case, the diffusion distance of carbon atoms from bainitic ferrite to adjacent film-like austenite increases in the isothermal process, resulting in the longer finishing time of bainite. More carbon atoms are retained in untransformed austenite after the transformation, forming more blocky retained austenite with low mechanical stability. The slight decrease in plasticity and impact toughness of the tested steel can be attributed to the increase in volume fraction of the retained austenite in the bainite microstructure.

The final microstructure of ultra-fine bainitic steel is composed of bainitic ferrite and retained austenite when the austempering temperature is above *M*s. Like the case of austenitizing temperature, the transformation kinetics, microstructural size and mechanical properties of bainitic steel is determined by the degree of supercooling. Although the incubation period is also not very obvious, and the finishing time of bainite for the 350 °C austempered sample is comparable to that of the sample austempered at 340 °C, the max transformation rate for the former occurs 18 s after the beginning of the austempered process (Figure 3c). A lower degree of supercooling means a lower driving force for the following bainite transformation, leading to a lower degree completion of the final transformation. Not only did the thickness of bainitic ferrite plates increase (Figure 10), but the volume fraction of the retained austenite also increased while the average carbon content decreased (Figure 11). On the condition that the volume fraction and carbon content of blocky retained austenite in the two samples are not much different, the volume fraction of film-like retained austenite in the 350 °C austempered sample is higher and the carbon content is lower compared to the 350 °C austempered sample. The TRIP effect tends to occur in film-like retained austenite with lower carbon content during tensile test to release stress concentration and prevent microcrack propagation, which results in a higher plasticity of the 350 °C austempered sample.

It has been known that the surface or tips of the prior martensite act as the nuclei for subsequent bainitic ferrite plates and accelerate the bainite transformation [51]. However, the finishing time of bainite in the paper increased when the austempering temperature was below *M*s, and the lower the austempering temperature, the longer the finishing time (Figure 3b). The phenomenon can be explained as follows. Firstly, the prior martensite divides the supercooled austenite grain and suppresses it as the bainitic ferrite grows. Secondly, more carbon atoms are ejected from prior martensite to untransformed austenite and improve the stability of the latter. And thirdly, decreasing the austempering temperature also strengthens the stability of supercooled austenite. The combined effect of all the above factors suppresses the growth of bainite, thus increasing the finishing time of bainite transformation.

As demonstrated by the aforementioned SEM and TEM micrographs, on the one hand, the introduction of prior martensite refines the bainitic ferrite plates and improves the fine grain strengthening effect. On the other hand, the presence of hard martensite grain can also improve the strength of the sample. The lower the austempering temperature, the higher the strength of the ultra-fine bainitic steel (Figure 14a). Furthermore, the size/morphology and carbon content of retained austenite are two major factors affecting the mechanical properties of ultra-fine bainitic steel: a finer grain size and a higher carbon content give rise to higher stability [52]. The prior martensite also refines the size of film-like retained austenite, and improve the carbon content within it (Table 2). The higher the carbon content of the film-like retained austenite, the higher the mechanical stability, and a more difficult TRIP effect occurs when the crack spreads to the austenite during deformation. Compared to that in the 320 °C austempered sample, more of the prior martensite formed in the 310 °C austempered sample, resulting in a lower completion degree of bainite transformation. However, the carbon content of film-like retained austenite in the latter sample is significant, so the impact toughness of the sample clearly improved.

A competitive relationship is observed between the nuclei sites and the growth of bainite sheaves when studying the effect of transformation temperature on bainite transformation kinetics, microstructural parameters, tensile and impact properties. The nuclei sites of bainite determine the thickness of bainitic ferrite plates and thus the strength of ultra-fine bainitic steel. The degree of transformation completion, which depends on the growth of bainite sheaves, not only determines the finishing time of bainite transformation, but also determines the plasticity and impact toughness of ultra-fine bainitic steel by affecting the volume fraction of film-like and blocky retained austenite and carbon content within it. Based on the above experimental results and discussion, the optimal treatment for medium-carbon ultra-fine bainitic steel in the present paper is by austenitizing it at 1000 °C and austempering it at 340 °C.

## 5. Conclusions

The effects of transformation temperature, i.e., austenitizing temperature and austempering temperature, on transformation kinetics, microstructural completion and parameters and the mechanical properties of medium-carbon ultra-fine bainite steel were investigated. The main conclusions are summarized as follows:

(1) With the austenitizing temperature increase from 950 to 1000 °C, the increase in the mechanical property of medium-carbon bainitic steel can be attributed to the higher volume fraction of bainite ferrite and the higher dislocation density within it. When the austenitizing temperature exceeds 1000 °C, the volume fraction of blocky retained austenite in the final bainite microstructure increases due to the obvious coarsening of prior austenite grain, and the plasticity and impact toughness are gradually reduced.
(2) The finishing time and completion degree of bainite transformation depend on the degree of the undercooling of prior austenite when the austempering temperature is above *M*s. With the increase in austempering temperature, the finishing time of bainite transformation is clearly extended, and the completion degree of bainite transformation decreases. The increment in elongation and the decrement in impact toughness for medium-carbon bainitic steel can be attributed to the coarsening of bainitic ferrite plates, the increase in the volume fraction of retained austenite with film-like morphology and the decrease in the carbon content within it.
(3) The prior martensite forms before bainite transformation when the austempering temperature is below *M*s. With the decrease in austempering temperature, the higher degree of supercooling and the presence of prior martensite refines the bainitic ferrite plates and increases the carbon content of film-like retained austenite, thereby significantly improving the strength and impact toughness of medium-carbon bainitic steel. However, the introduction of prior martensite inhibits the growth of bainite sheaves, and thus reduces the completion of bainite transformation, resulting in a significant increase in the volume fraction of blocky retained austenite and a slight decrease in the elongation of medium-carbon bainitic steel. Based on the experimental results and discussion, the optimal treatment for medium-carbon ultra-fine bainitic steel in the present paper is through austenitization at 1000 °C and austempering at 340 °C.

## Figures and Tables

**Table 1:** Microstructural parameters of the tested steels at different austenitizing temperatures.

Temperature, °C	*ρ* × 10^15^ m^−2^	V*_B_*, %	V*_γ_*, %	C*_γ_*, wt. %	V_F-RA_, %	C_F-RA_, wt.%	V_B-RA_, %	C_B-RA_, wt.%
950	3.3 ± 0.5	83.9 ± 0.6	16.1 ± 0.4	1.3 ± 0.1	8.5	1.4	7.6	1.2
1000	5.4 ± 0.3	91.2 ± 0.5	8.8 ± 0.5	1.9 ± 0.3	3.4	2.5	5.5	1.3
1050	4.9 ± 0.7	89.6 ± 0.5	10.4 ± 0.5	1.8 ± 0.4	3.5	2.4	6.9	1.2
1100	4.1 ± 0.8	86.6 ± 0.6	13.4 ± 0.4	1.5 ± 0.3	4.5	1.6	8.9	1.4

**Table 2:** Microstructural parameters of retained austenite from the samples austempered at different temperatures.

Temperature, °C	V_F-RA_, %	C_F-RA_, wt.%	V_B-RA_, %	C_B-RA_, wt.%
310	2.5	2.4	12.9	1.5
320	9.4	1.7	6.9	1.5
340	3.4	2.5	5.5	1.4
350	7.7	1.7	6.7	1.4

## Data Availability

Data are contained within the article.
